# On the Use of Looped Wire in the Removal of Foreign Bodies from the Air-Passages, with a Report of Two Cases

**Published:** 1861-02

**Authors:** J. J. Tomson

**Affiliations:** Davenport, Iowa


On the use of Looped Wire in the Removal of Foreign Bodies from the Air-Passages, with a Report of Two Cases. *By J. J. Tomson, M. D.*, of Davenport, Iowa.—Some time in the month of May last, a lad about eight years old, whose parents reside in this city, accidentally inhaled into the trachea a piece of clay pipe-stem, about one and a half inches long, and of large size. Dr. Maxwell saw the patient, and used the probang, hoping thereby to dislodge the foreign body and enable the boy to cough it up. After using the probang, with some other means, the boy was relieved and it was hoped that he had coughed the pipe-stem up, and perhaps swallowed it into the stomach. He was quite relieved for some six days, running and playing as usual.

On the sixth day after inhaling the pipe-stem, one of his playmates threw a stone which accidentally struck him upon the back. From this time he became rapidly worse, with all the symptoms of a foreign body within the air passages. A council of physicians was called, who agreed that there was a foreign body in the trachea, and that an operation was the only probable means of relief to the boy. The operation was performed by Dr. Adler, assisted by Drs. Baker, Maxwell, Fountain, and myself. After the operation, a variety of instruments and means were used, which were not successful in removing the foreign body. In the afternoon of the same day, and the morning following, Drs. Adler and Maxwell made other attempts with no better success. On the afternoon of the second day, about thirty-six hours from the time of the operation, and more than one week from the time of the inhalation of the pipe-stem, I was requested by Dr. Adler to visit the patient, with himself and Dr. Maxwell. The patient was rapidly failing, and we felt that he would certainly succumb, unless the foreign body was soon removed. After trying the forceps, hooks, etc., I suggested the use of a *looped wire.* A piece of small wire, about two feet long, was obtained, and looped in the middle, of sufficient size to embrace the end of the pipe-stem (on the same principle as removing corks from a bottle with a string.) The patient's head being well thrown back, I proceeded to introduce the *looped wire.* On passing it down to the right bronchus, it came in contact with the foreign body. At this point, I raised the end which I held in my hand and pressed the end next the foreign body back toward the spine, so as to pass my wire behind the pipe-stem. The pipe-stem was firmly impacted in the bronchus, so that it required some force to push the wire between it and the walls of the bronchial tube. The wire was passed some two inches or more below the point of obstruction, and then, on gently withdrawing it, the loop came in contact with the lower end of the pipe-stem, which was thus easily removed. The orifice of the trachea was closed, and the boy made a rapid recovery.

On the twenty-fifth of last month, my partner, Dr. Maxwell, and myself were sent for by Dr. Carpenter, of Blue Grass, to assist him in removing a grain of corn from the trachea of a child about one year old. The operation was performed by Dr. Maxwell: after which I passed the loop of wire as in the other case. It was passed down the right bronchus, and passed quite easily the point of obstruction; and on its removal, it brought the kernel of corn into the trachea, which soon after made its appearance at the orifice, and was easily removed.

I wish to call the attention of the profession to this *simple, cheap*, and *harmless* instrument, from the fact that I believe it will succeed in some cases where nothing else will. It can be used with perfect freedom by any one who is acquainted with the anatomy of the lungs, in searching far into the air-passages for small bodies, with little or no risk of producing serious irritation. There are other cogent reasons for its trial which will suggest themselves to the mind of every medical man. I submit its trial, with the cases above reported, to the profession, hoping that it may be found of *some* service in such painful and unfortunate cases.—*Am. Med. Times.*

